# Identification of a novel *GLA* mutation (Y88C) in a Korean family with Fabry nephropathy: a case report

**DOI:** 10.1186/s12881-016-0338-7

**Published:** 2016-10-24

**Authors:** Yosep Chong, Minyoung Kim, Eun Sil Koh, Seok Joon Shin, Ho-Shik Kim, Sungjin Chung

**Affiliations:** 1 Department of Hospital Pathology, College of Medicine, The Catholic University of Korea, Seoul, Republic of Korea; 2 Department of Internal Medicine, College of Medicine, The Catholic University of Korea, Seoul, Republic of Korea; 3 Department of Biochemistry, College of Medicine, The Catholic University of Korea, Seoul, Republic of Korea

**Keywords:** α-galactosidase A, Dialysis, Fabry disease, Kidney biopsy, Proteinuria

## Abstract

**Background:**

Fabry disease is a rare X-linked lysosomal storage disorder caused by α-galactosidase A deficiency. With the advancement of molecular diagnostic tools, more disease-causing mutations in α-galactosidase A (*GLA*) have been identified in Fabry disease. We found a novel mutation in a Korean family with predominant renal manifestations of the disease.

**Case presentation:**

A 24-year-old man who wanted to donate a kidney to his 28-year-old brother with end-stage renal disease of unknown cause was evaluated. The 24-year-old man underwent percutaneous renal biopsy because of an accidentally found proteinuria. Electron microscopy of his renal biopsy showed numerous electron-dense multi-lamellar inclusions in the epithelial cytoplasm, typical for Fabry disease. Clinical and laboratory evaluation including the assessment of GLA enzyme activity and direct DNA sequencing in four members of the family were performed. Renal biopsy findings in the two affected male patients were described. Re-evaluation of a renal biopsy specimen of his 28-year-old brother obtained when he was diagnosed with renal failure revealed a very focal area of suspicious multilamellated structures in the Bowman’s space. DNA sequencing on the young man, his brother, and his mother revealed a novel GLA gene mutation, c.263A > G (p.Tyr88Cys). The three all showed decreased α-galactosidase A activity.

**Conclusion:**

A novel *GLA* mutation, c.263A > G (p.Tyr88Cys), was found in a Korean family with predominant renal manifestations of Fabry disease.

## Background

Fabry disease (FD, OMIM#301500) is a rare, progressive, X-linked, and multisystemic disorder characterized by α-galactosidase deficiency resulting from mutations in the α-galactosidase (*GLA*, OMIM*300644) gene at Xq22.1 [1, 2]. The enzyme α-galactosidase A (GLA) is a ubiquitous lysosomal acid hydrolase. Its deficient activity can lead to progressive accumulation of globotriaosylceramide and other glycosphingolipids within the lysosomes of various cell types throughout the body, causing severe and potentially life-threatening target-organ complications such as cardiovascular diseases, stroke, renal failure, and eventually death [2].

The spectrum of clinical presentations in male patients with FD varies widely, from severe classic phenotype to relatively mild late-onset variant form [2, 3]. According to previous reports, the clinical manifestations in the classic form of FD starting from childhood or adolescence include severe symptoms such as acroparesthesia, hypohidrosis, corneal opacities, stroke, cardiac abnormalities, and renal disorders with high mortality [3–5]. On the contrary, the clinical manifestations of the variant form comprising almost 70 % of FD patients [6] are usually mild and limited to the heart or kidney [3, 4]. Due to mild symptoms, these patients are often found after disease progression. They only show findings of focal segmental glomerular sclerosis. For such cases, the key pathognomonic features such as electron-dense multi-lamellar inclusions can be obscured by extensive sclerosis on renal biopsy [4, 6]. As a result, it can be difficult to diagnose FD in time, particularly its variant form which is often undiagnosed or misdiagnosed as other heart or kidney diseases [4].

Recently, there have been amazing advances in molecular diagnostics for FD. More than 700 mutations in *GLA* gene have been identified, including missense mutations, small deletions/insertions, splice mutations, and large gene rearrangements [7]. These are listed in the Human Gene Mutation Database (http://www.hgmd.cf.ac.uk/), Fabry Database (http://fabry-database.org/), and ClinVar (http://www.ncbi.nlm.nih.gov/clinvar/). New mutations are still being discovered.

In the present study, we identified a novel mutation, Tyr88Cys, in *GLA* in a Korean family with late-onset FD variant. We also discussed their clinicopathologic feautures and the molecular diagnosis of FD.

## Case presentation

### Subjects and kidney biopsy procedure

We studied four members of a Korean family. Clinical and physical examinations were performed for each member. All family members gave written consent before we obtained blood and kidney samples from them.

Percutaneous renal biopsy was done by nephrologists under ultrasonographic guidance using an automated biopsy gun as previously described [8]. Histopathologic diagnosis on each sample was made comprehensively based on all clinical data and pathologic findings.

### Analysis of genomic DNA and GLA enzyme activity

Genomic DNA samples were isolated from peripheral white blood cells using QIAamp DNA Blood Mini Kit (Qiagen, Hilden, Germany). For sequencing, samples were pooled in an equimolar ratio. Genomic DNA library (10 pM) containing 1 % PhiX control library were prepared and sequenced on a MiSeq platform (Illumina, San Diego, CA) using MiSeq v2 chemistry. Next generation sequencing (NGS) data analyses were performed using bioinformatics pipeline of Labgenomics Co. Ltd. (Gyeonggi-do, Republic of Korea). Briefly, the paired-end sequencing reads of 250 bases in length were binned for each sample according to the index and mapped to the human genome assembly (hg19). Burrows-Wheeler Aligner (BWA) was used to align these sequences to the human genome allowing up to 2 base mismatches (RefSeq NM_000169). Pathogenic variants and variants of unknown significance (VUS) detected by NGS were confirmed by Sanger sequencing analyses with AB 3730XL DNA Analyser (Life Technologies, Carlsbad, CA, USA) in accordance with the manufacturer's instructions. Sequencing results were searched against data of Korean exomes obtained from Labgenomics, data from Korea National Institution of Health (KNHIH), dbSNP, and 1000 Genomes Project databases. To determine whether the newly discovered mutation in *GLA* might contribute to FD, prediction tests including Polyphen-2 and MutationTaster were used as described previously [9–11].

For all subjects, we assessed lysosomal GLA enzyme in the leukocytes by fluorometric assay using substrate 4-MU-α-D-galactopyranoside [12, 13]. Enzyme level was expressed as nmole of cleaved substrate per milligram of protein per hour at 37 °C. The enzyme activity was normalized to the activity of the control (100 %).

## Results

**Fig. 1:**
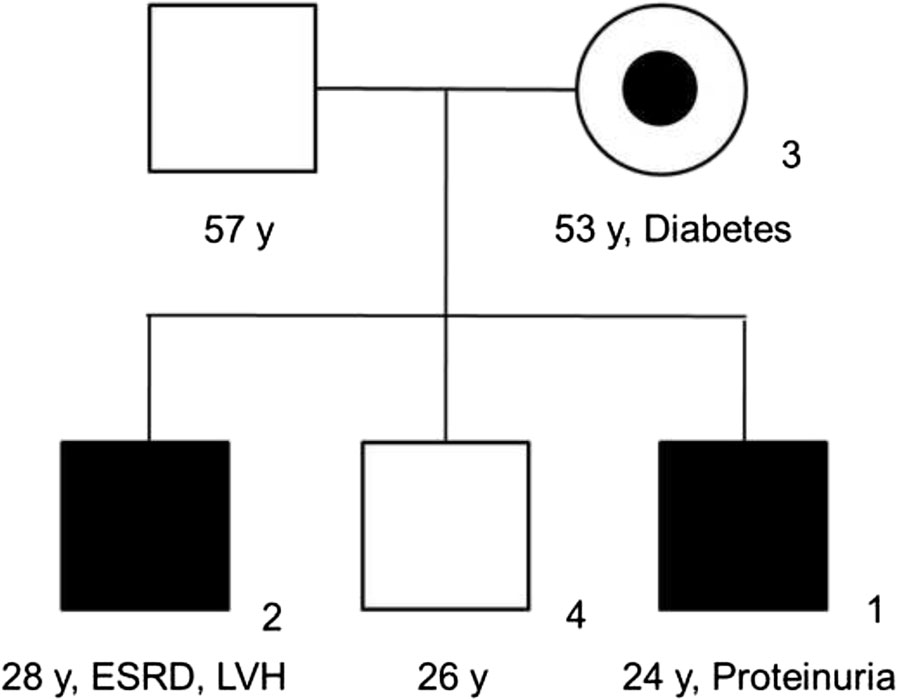
The family pedigree. The shaded squares indicate affected men (patients 1 and 2). The circle with a dot indicates heterozygous woman (patient 3). ESRD, end-stage renal disease; y, years

The clinicopathologic features of these patients including enzymatic and genetic analysis results are summarized in Table 1. The pedigree of the family is shown in Fig. 1. A 24-year-old man (patient 1) was evaluated voluntarily to donate a kidney to his 28-year-old brother (patient 2) who had end-stage renal disease (ESRD) and had been undergoing peritoneal dialysis for the previous 2 years. Patient 1 had an unremarkable medical history without medications. His assessment was overall satisfactory except a proteinuria of 871 mg/day (reference range, < 150 mg/day) that was discovered incidentally. A kidney biopsy was performed in view of the unaccountable proteinuria.

**Table 1:** Clinicopathologic features of the four patients described in this case report

Subject No.	Age/Sex	Clinical findings	α-galactosidase A	c.263A > G mutation	Kidney biopsy findings
Patient 1	24/M	Proteinuria	Decreased (2.2 nmol/hr/mg protein)	Present	Consistent with FD
Patient 2	28/M	End-stage renal disease, hypertension, left ventricular hypertrophy	Decreased (4.0 nmol/hr/mg protein)	Present	Consistent with FD
Patient 3	54/F	Mild proteinuria, diabetes	Slightly decreased (32.6 nmol/hr/mg protein)	Present	Unavailable
Individual 4	26/M	Unremarkable	Normal (43.4 nmol/hr/mg protein)	Not present	Unavailable

*FD* Fabry disease, *F* female, *M* male, No, number

In 2014, which was two years ago before patient 1’s visit, patient 2 (his brother) had presented at the age of 26 years with nephrotic-range proteinuria (24 h urine protein, 8486.4 mg/day) and renal insufficiency with serum creatinine of 8.06 mg/dL (reference, 0.50–1.20 mg/dL). At that time, a renal biopsy was performed and the finding was glomerulosclerosis with moderate to severe tubulointerstitial fibrosis indicating advanced renal failure. As a result, plans to initiate dialysis were immediately made. Considering patient 1’s proteinuria, there was ample room for doubt regarding familial kidney disease. Accordingly, patient 2’s previous renal biopsy slides and medical records were re-reviewed.

The mother (patient 3) of patients 1 and 2 was a 53-year-old female who had been diagnosed with diabetes 10 years earlier. She had been completely asymptomatic. However, based on her sons’ results, she also underwent screening. Results revealed normal renal function with slightly elevated proteinuria (154.8 mg/day). Renal biopsy in patient 3 was not performed. Individual 4, the second son of patient 3, showed normal results for all laboratory findings, including renal function.

Apart from renal involvement, the affected patients (1, 2, and 3) had no other clinical manifestations such as peripheral neuropathy, angiokeratoma of skin, corneal opacities, abdominal pain, diarrhea, or arrhythmias. Patient 2 only showed high blood pressure (165/100 mmHg) and left ventricular hypertrophy on echocardiographic examination.

**Fig. 2:**
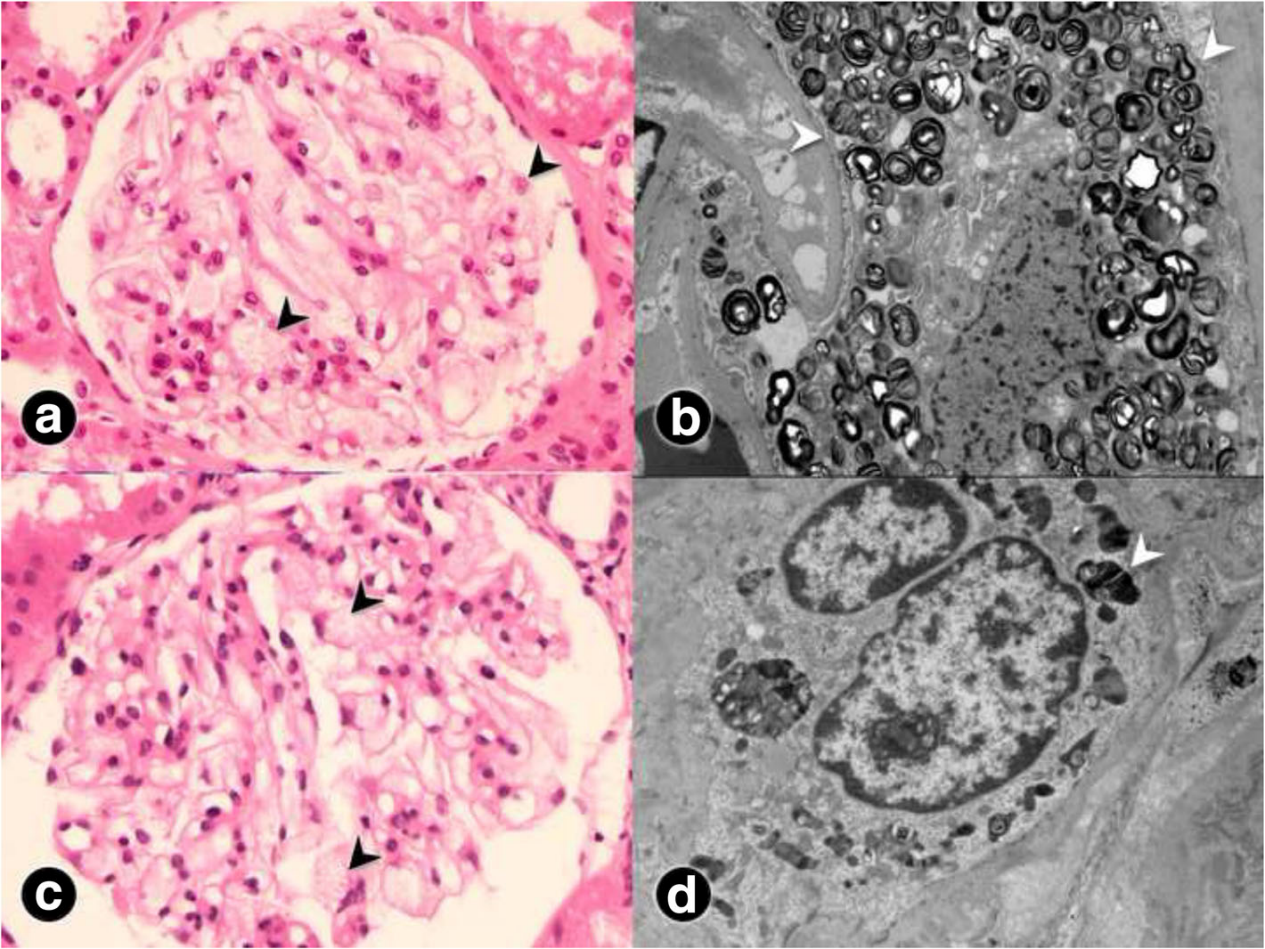
Light and electron microscopic findings of renal biopsies from patients 1 and 2. **a** On microscopic examination of patient 1, there are some vague vacuolizations of podocytes (black arrowhead), although he showed normal renal function and proteinuria (H&E stain, ×400). **b** Multilamellated myelin figures (white arrowhead), so-called zebra bodies—which are typical findings in Fabry disease—are found in patient 1’s podocytes on electron microscopy. **c** Similar vacuolated epithelial cells (black arrowhead) are found in the relatively preserved glomerulus of patient 2, whereas most glomeruli are damaged by sclerosis (H&E stain, ×400). **d** A focal area showing a tiny, dark–stained, round multilamellar inclusion (white arrowhead) on the electron microscopy result for patient 2

Renal biopsy from patient 1 revealed 12 glomeruli with only a subtle change on microscopic examination. However, nothing was significant (Fig. 2a). The glomerular epithelial cells were swollen and showed somewhat bubbly appearance with vague vacuoles. The renal tubules, interstitium, and blood vessels were unremarkable. There was no immune deposition on immunofluorescence for immunoglobulins. Electron microscopy revealed typical electron-dense multi-lamellar inclusions and zebra bodies in the cytoplasm of the epithelial cells (Fig. 2b).

With the suspicion of a familial disease, we punctiliously reviewed patient 2’s renal biopsy results. His biopsy specimen contained 9 glomeruli which showed 7 global and segmental glomeruloscleroses. There was moderate to severe tubulointerstitial damages, including mononucleocyte and neutrophil infiltration, interstitial fibrosis, and tubular atrophy. There was no immune deposition on immunofluorescence staining. However, detailed observation of the unaffected glomeruli revealed characteristic vacuolization of epithelial cells (Fig. 2c), which had been missed 2 years ago due to profound glomerular damage. On electron microscopy, there was a very focal area with suspicious multilamellated structures in the Bowman’s space (Fig. 2d). These findings in patients 1 and 2 were consistent with Fabry nephropathy.

**Fig. 3:**
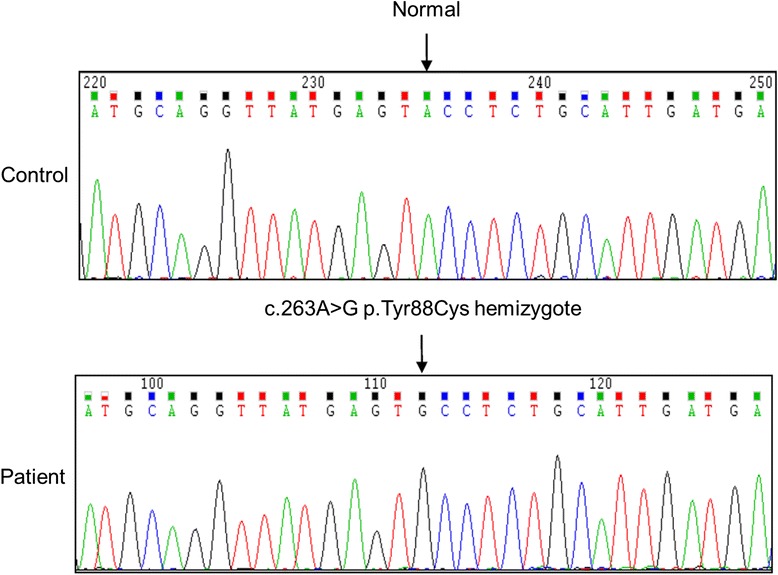
Electropherogram of exon 2 of α-galactosidase A in this family with Fabry nephropathy. The analysis shows a mutation of A > G at nucleotide 263, resulting in an amino acid substitution p.Tyr88Cys

DNA sequencing for *GLA* showed that patients 1, 2, and 3 carried a c.263A > G (p.Tyr88Cys) mutation in exon 2 (Fig. 3). This mutation has not been previously reported in Genbank or other mutation database of FD. Genetic analysis in individual 4 was unremarkable. To confirm the potential role of this mutation, we used tools to predict the possible impact of an amino acid substitution on the structure of a protein for further analysis. The Polyphen-2 scores for the Tyr88Cys mutation in *GLA* ranged from 0.990 to 1.000, suggesting that the mutation was likely to damage the protein. The score for amino acid changes and the probability value drawn by MutationTaster were 194 and 0.999 (the prediction was highly secure), respectively. These results strongly suggest that the novel mutation might have a damaging effect on the enzyme’s structure.

From our determination of the GLA enzyme in blood leukocytes, patient 1 showed a decreased GLA protein level (at 2.2 nmol/h/mg, average of 1,000 normal controls: 62.3 nmol/h/mg; reference: 35–100 nmol/h/mg) with decreased GLA activity (23.1 %, average of 1,000 normal controls: 92.0 %; reference: 85–95 %), confirming enzymatic defect of GLA. Protein level and activity of GLA in patient 2 were 4.0 nmol/h/mg protein (average of 1,000 normal controls, 66.8 nmol/h/mg) and 22.3 % (average of 1,000 normal controls, 93.2 %), respectively. Patient 3 was thought to be a healthy female carrier. However, her GLA protein level and activity were 32.6 nmol/h/mg protein (average of 1,000 normal controls, 79.0 nmol/h/mg) and 89.6 % (average of 1,000 normal controls, 92.3 %), respectively, which were below normal ranges, indicating a heterozygous state. The protein level and activity of GLA enzyme in individual 4 were 43.4 nmol/h/mg protein (average of 1,000 normal controls, 61.6 nmol/h/mg) and 94.0 % (average of 1,000 normal controls, 93.2 %), respectively, which were within normal ranges.

## Conclusion

In this report, we identified a new GLA mutation (c.263A > G p.Tyr99Cys) in a Korean family with predominant renal involvement of late-onset FD. We have deposited this new mutation data to the Fabry Database (http://fabry-database.org/, ID: 329). Although these family members shared the same GLA mutation, substantial differences in clinicopathologic features and residual enzyme activities were noticed, which can be potential pitfall for proper early diagnosis of FD, especially for its late-onset variant.

*GL*A mutation in coding sequence can result in amino acid changes, which is likely to alter protein conformation and subsequent functional defect of the GLA enzyme [14]. The 88 Tyr amino acid belongs to the group of amino acids buried in the hydrophobic core likely involved in protein folding [14, 15]. Although the current study did not cover the functional pathogenesis of Tyr88Cys, the potential deleterious effect of the Tyr88Cys mutation was predicted in this study using bioinformatics tools Polyphen-2 and MutationTaster. However, further study on the functional alteration resulted from Y88C mutation is still needed.

The genotype-phenotype correlation is not perfect in FD [5]. In this report, two brothers (patients 1 and 2) were only 4 years apart in age. However, they demonstrated somewhat different clinical courses. Patient 2 was already on peritoneal dialysis due to resultant ESRD while patient 1 only had mild proteinuria with normal renal function, although they had similar levels of GLA. This supports the notion that environmental and other genetic factors can affect the disease severity and phenotypic variability of FD [5]. This is consistent to the findings of a previous study showing marked intra-familial variability in residual GLA enzyme activity and the natural disease course of FD [16]. The GLA activity in patient 3, the mother, was slightly below the normal range without evident clinical symptoms. Female carriers of *GLA* mutations in FD have been considered as asymptomatic originally. However, it is now accepted that heterozygous females may also develop mild to severe clinical manifestations of FD [5, 17]. The characteristic presentations including delayed onset and a higher probability of a single organ involvement have been thought to be due to random X chromosome inactivation [18, 19].

Recently found novel GLA mutations are mainly from patients with mild and late-onset FDs [13, 20]. Characteristically, late-onset FD has renal or cardiac involvement without characteristic skin lesions or pain crises. FD is often diagnosed when patients are in their 50 s or 60 s [11].

This report shows an important point regarding kidney biopsy. In patient 1 who had only proteinuria, biopsy played a valuable role in diagnosing the FD missed in his family member. Although his kidney specimen demonstrated almost normal appearance in light microscopy examination, the electron microscopy clearly demonstrated lamellated myelin structures and confirmed the diagnosis of typical FD. On the contrary, we missed the FD findings in patient 2’s kidney biopsy 2 years ago due to extensive sclerotic glomerular damage. The lamellated structure covered on the glomerular surface may inhibit glomerular filtration and result in endothelial injury [15]. Subsequently, endothelial cells in the vicinity of any damaged podocytes are submitted to additional hydraulic forces, leading to glomerular collapse and sclerotic change [15]. This diffuse and progressive destruction of the kidney structure may make it difficult to recognize the typical FD findings. Thus, it is important to secure the minimum requirement of preserved glomeruli to obtain accurate pathologic diagnosis. For female patients 3 with minimal proteinuria, it might be necessary to perform renal biopsy in order to identify whether the proteinuria was due to FD or diabetes. The majority of female carriers do not develop renal disease with glycosphingolipids deposition. However, some clinically normal heterozygous females have typical kidney biopsy findings [21].

Early diagnosis of FD is crucial for commencing early enzyme replacement therapy and improving the natural disease course by preventing progressive organ failure [22]. Although diabetes, hypertension, and chronic glomerulonephritis are the most common causes of ESRD, the causes of primary renal disease in more than 20 % of patients with ESRD are still uncertain [23, 24]. Therefore, it is reasonable to bestow some consideration on screening for FD in patients with renal failure of unknown causes at an early age. In male patients, the diagnosis of FD can be made based on markedly deficient GLA enzyme activity in plasma or leukocytes and GLA mutations [9]. In order to identify affected females, it is important to combine biochemical assays with molecular analyses of the *GLA* gene [16, 17]. Considering that FD has progressive effects on various organs, vital organ damage would have already begun regardless of the activity of GLA enzyme. This implies that biopsy of the involved organ could help us identify the extent of FD and start therapy accordingly.

Because the parents of patient 3 have already passed away with unspecified causes at the ages of 62 and 67 years, respectively, it is impossible to know at which generation that this mutation started in this family. However, patient 3 had seven siblings. Her two older brothers died of renal disease that required dialysis at the ages of 31 and 57 years, respectively. Molecular testing for *GLA* gene for patient 3’s youngest brother was normal. Tests for other siblings are now being carried out. Past history of her two brothers suggests the possibility that the patient 3’s mother was a carrier of the mutant *GLA* gene. However, it is also possible that this mutation is a *de novo* mutation or that either the mother or the father of patient 3 is a germ line mosaic for the mutation [25, 26]. In patients 1 and 2, the enzymatic activity of GLA needs to be tested serially so that treatment can be started before other organ involvement becomes irreversible. The female carrier patient 3 also needs to be followed up closely for the possibility of her developing FD-related symptoms. A request for enzyme replacement therapy has been submitted to The Health Insurance Review and Assessment Service.

In summary, this report revealed the presence of a c.263A > G p.Tyr88Cys mutation in exon 2 of *GLA* gene in a Korean family with Fabry nephropathy. The detection of this new mutation in *GLA* highlights the notion that there is no single predominant mutation in FD. This report also described the possibility that distinctive findings of Fabry nephropathy might not appear clearly in severely damaged kidneys.

## Abbreviations

4-MU-α-D-galactopyranoside: 4-Methylumbelliferyl-α-D-galactopyranoside
Cys: Cysteine
dL: Deciliter
DNA: Deoxyribonucleic acid
ESRD: End-stage renal disease
FD: Fabry disease
GLA: α-galactosidase A
H&E: Hematoxylin and eosin
hr: Hour
kb: Kilobase
mg: Milligram
mmHg: Millimeters of mercury
nmol: Nanomole
Tyr: Tyrosine
